# AGE, SEX, AND FRAILTY INFLUENCE AGE-DEPENDENT CHANGES IN VENTRICULAR STRUCTURE AND FUNCTION IN C57BL/6 MICE

**DOI:** 10.1093/geroni/igz038.338

**Published:** 2019-11-08

**Authors:** Susan E Howlett, Alice Kane, Elise Bisset, Kaitlyn Keller

**Affiliations:** 1 Dalhousie University, Halifax, Canada; 2 Paul F. Glenn Center for Biology of Aging Research at Harvard Medical School, Boston, Massachusetts, United States

## Abstract

The heart undergoes maladaptive changes during aging that set the stage for cardiovascular diseases, but frail older individuals are most likely to develop such diseases. We investigated the impact of frailty on left ventricular (LV) remodeling in male and female mice (aged 9-23 mos). Ventricular function/structure and frailty were assessed with echocardiography (Vevo 2100) and a frailty index (FI) tool. Fractional shortening (systolic function) increased with age (9 vs 23 mos) in males (27.7±2.6 vs 38.4±1.6%; p<0.05) and females (26.9±1.4 vs 32.5±1.8%; p<0.05); similar results were seen with ejection fraction. Conversely, E/A ratios (diastolic function) declined with age in males (1.9±0.1 vs 1.3±0.1; p<0.05) and females (2.1±0.3 vs 1.6±0.1; p<0.05). LV mass and LV internal diameter (diastole) increased with age in females but not in males, while intraventricular septum (diastole) increased in males only. As age-dependent changes were heterogeneous, we stratified the data by FI scores. Interestingly, fractional shortening (r=0.52; p=0.006) and ejection fraction (r=0.52; p<0.0001) were graded by FI score, but only in males. By contrast, LV mass was graded by frailty, but only in females (r=0.55; p<0.0001). Thus, diastolic function declines with age in both sexes while systolic function actually increases. Aging females display increased LV mass and LV dilation whereas older males exhibit septal thickening. These maladaptive changes are graded by frailty, suggesting that cardiac aging is prominent in those with poor overall health. Age, sex and frailty influence cardiac aging, which may predispose frail older men and women to develop different cardiovascular diseases as they age.

